# Focal spleen lesions in loiasis: A pilot study in Gabon

**DOI:** 10.1371/journal.pntd.0012448

**Published:** 2024-08-23

**Authors:** Bayode R. Adegbite, Federico G. Gobbi, Cristina Mazzi, Fabrice Beral M’Baidiguim, Anita Lumeka, Andréa R.O Obele Ndong, Jean R. Edoa, Yabo J. Honkpéhèdji, Jeannot F. Zinsou, Jean C. Dejon-Agobé, Rella Zoleko-Manego, Michael Ramharter, Ayola A. Adegnika, Francesca Tamarozzi

**Affiliations:** 1 Centre de Recherches Médicales de Lambaréné (CERMEL), Libreville, Gabon; 2 Institut für Tropenmedizin, Eberhard Karls Universität Tübingen, Tübingen, Germany; 3 Department of Infectious-Tropical Diseases and Microbiology, IRCCS Sacro Cuore Don Calabria Hospital, Negrar di Valpolicella, Verona, Italy; 4 Department of experimental and clinical sciences, University of Brescia, Brescia, Italy; 5 Clinical Research Unit, IRCCS Sacro Cuore Don Calabria hospital, Negrar di Vapolicella, Verona, Italy; 6 Center for Tropical Medicine, Bernhard Nocht Institute for Tropical Medicine & I Dep of Medicine University Medical Center Hamburg-Eppendorf, Hamburg, Germany; 7 German Center for Infection Research, Partner Site Hamburg-Lübeck-Borstel-Riems, Hamburg, Germany; 8 German Center for Infection Research, Partner Site Tübingen, Tübingen, Germany; Seoul National University College of Medicine, REPUBLIC OF KOREA

## Abstract

**Background:**

Infection with the filarial nematode *Loa loa*, endemic in Central and Western Africa, has been associated with increased morbidity and mortality. A number of reports described the presence of spleen nodules, originating from degenerating microfilariae, in humans and animals infected with *L*. *loa*. The long-term consequences of this process on individuals chronically exposed to infection in terms of spleen function and possible link with excess mortality are unknown. The aim of this study was to evaluate the prevalence of focal spleen lesions, their evolution over time, and markers of spleen function, in individuals with *L*. *loa* infection living in highly endemic areas of Gabon.

**Methodology/principal findings:**

This was a cross-sectional study followed by a longitudinal study of the subset of individuals with spleen nodules. Two hundred sixteen participants from Ngounié and Moyen-Ogooué provinces of Gabon, reporting a history of eyeworm migration and/or Calabar swelling, were included. Participants were categorized into infected microfilaraemic with low (N = 74) and high (N = 10) microfilaraemia, and symptomatic amicrofilaraemic (N = 132), based on blood microscopy. Howell-Jolly bodies in erythrocytes, as indirect marker of spleen functional impairment, were within normal ranges. On ultrasound, no evident signs of spleen fibrosis or hypotrophy were observed. Multiple spleen hypoechoic centimetric macronodules were observed in 3/216 participants (1.4%), all with microfilaraemic *L*. *loa* infection (3.4% of microfilaraemics); macrondules disappeared at the 6-months follow-up examination in 2/3 individuals. Spleen hypoechoic micronodules, persisting at the 6-months follow-up, were detected in 3/216 participants (1.4%), who were all amicrofilaraemic.

**Conclusions/significance:**

Transitory spleen macronodules are present in a small but consistent proportion of individuals with microfilaraemic loiasis, appearing a rather benign phenomenon in terms of impact on spleen morphology and function. Their occurrence should be taken into consideration to avoid misdiagnosis and mistreatment. Prevalence and significance of spleen micronodular ultrasound patterns in the general population would be also worth evaluating.

## Author summary

*Loa loa* is a vector borne, filarial worm affecting an estimated 20 million people in Central and West Africa. Long considered a benign infection, more recently it has been associated with substantial morbidity and increased mortality in individuals with high *L*. *loa* microfilaraemia, prompted to reconsider the importance this infection and investigate the potential pathophysiological mechanisms. The spleen has a central role in the response to pathogens, including parasites; therefore, its dysfunction may lead to an increase in the risk and severity of infectious diseases, which might contribute to the observed excess mortality in hypermicrofilaremic loiasis. The presence of spleen centimetric nodules, originating from degradation of microfilariae, in humans and animals infected with *L*. *loa*, has been described, but the long-term consequences of this process on individuals chronically exposed to infection in terms of spleen function are unknown. In this study performed in individuals with *L*. *loa* infection living in highly endemic areas of Gabon, we found a prevalence of 3.4% spleen nodules in microfilaraemic participants, while no such lesions were observed in symptomatic amicrofilaraemic participants. Nodules were transitory and appeared to be a rather benign phenomenon in terms of impact on spleen morphology and function.

## Introduction

Infection with the vector-borne filarial nematode *Loa loa* is endemic in Central and West Africa, with estimated 20 million people chronically infected in high or intermediate risk areas (i.e. having *L*. *loa* prevalence higher than 20%) [1]. The parasite is transmitted through the bite of *Chrysops* spp flies. Adult worms reside and move in intermuscular fascial layers and subcutaneous tissue, where females produce first-stage larvae (microfilariae; mf), which circulate in the blood during daytime. When symptomatic, the classical clinical presentation of loiasis include the migration of the adult worm under the conjunctiva (“eyeworm”), transient episodes of localized angioedema (“Calabar swelling”), persistent itching, arthralgia, and a wide range of other atypical and non-specific, including some potentially life-threatening, manifestations, as reviewed in [1,2].

Manifestations of loiasis have been reported as major reasons for people seeking medical consultation in endemic areas [3]; however, loiasis has been classically considered a benign condition. Attention to loiasis has been mainly driven by the severe neurological adverse events that may occur after administration of diethylcarbamazine (DEC) or ivermectin (IVM) in individuals with high *L*. *loa* mf load, which hampers Mass Drug Administration (MDA) programs for lymphatic filariasis and onchocerciasis in co-endemic areas [4]. Only recently, substantial morbidity [5] and increased risk of mortality associated with high *L*. *loa* microfilaraemia were found in patients with loiasis [6,7]. These findings prompted the medical and scientific community to reconsider the importance of this parasitic infection and investigate the potential pathophysiological mechanisms causing specific morbidity and mortality.

In the last century, a number of reports described the presence of spleen nodules in humans and animals infected with *L*. *loa*, corresponding histologically to inflammatory granulomas with necrosis and eosinophil infiltration around degenerated mf (reviewed in [8]). Spleen nodules in humans were reported to be caused also by *Wuchereria bancrofti*, but not other filarial species frequently affecting humans, such as *Mansonella perstans* (reviewed in [8]).

Clinically, the nodules seem transitory with evolution over about 1–2 years, and not always associated with high level microfilaraemia [8,9]. On imaging, they were described as hypoechoic on ultrasound; hypodense and hypovascularized on contrast-enhanced computed tomography; and hyperintense on T2-weighted and hypointense on T1-weighted magnetic resonance imaging, with a rim enhancement during the venous phase [8]. The recognition of loiasis among the causes of such lesions is important, since they may pose differential diagnostic problems with other infectious and proliferative diseases, exposing patients to unnecessary diagnostic procedures and even splenectomy [9,10].

Since over 50% of cases of spleen nodules in loiasis have been reported in the past decade in migrants attended in the Department of Infectious-Tropical Diseases and Microbiology (DITM) in Negrar, Italy, alone [8,9,11] it could be speculated that spleen nodules due to *L*. *loa* might be more common than generally thought but only rarely observed in single-time observations because of their transitory nature. Indeed, only a systematic imaging evaluation of all attended patients, as carried out at DITM, could have allowed documenting these transient asymptomatic lesions.

While it seems that splenic granulomas in loiasis originate from the process of degradation of mf in the spleen [12–15], it remains to be elucidated whether it is a “normal” mechanism or is the result of some less efficient process, making granulomas detectable only in some individuals. Furthermore, the long-term consequences of this process in individuals chronically exposed to infection are largely unknown. Indeed, since the spleen has a central role in the response to pathogens [16,17], its dysfunction may lead to an increase in the risk and severity of infectious diseases which—if confirmed—might be speculated to be causally linked to the observed excess mortality of hypermicrofilaremic loiasis. In a previous review [8], we could not find any study investigating the association between loiasis and incidence or severity of infections caused by encapsulated bacteria. In addition, the interaction between filarial nematodes and malaria in terms of clinical features was unclear; furthermore, no study so far specifically addressed the presence of functional hyposplenism in loiaisis or any other filarial infection.

The aim of this study was to evaluate the prevalence of spleen lesions, markers of spleen function, and the evolution of focal spleen lesions over time, in individuals with *L*. *loa* infection living in highly endemic areas of Gabon.

## Methods

### Ethics statement

The study was approved by the Ethics Review Committee of the Centre de Recherches Médicales de Lambaréné (CERMEL) (number CEI-013/2022). All participants of 18 years of age and above signed an informed consent form (ICF); parents/guardias signed the consent form in case of minors, with oral assent from the minor. The presence of a witness knowing the person was required in case that the identity of the participant could not be assured by an appropriate document. An impartial witness was required to assist the informed consent process in case of illiteracy of the study participant. Identifying data of each participant were recorded in the ICF, coupled with the unique participant ID used to identify all study data in a pseudonymized manner. All analyses were carried out identifying the participant by the unique ID code.

### Study design and objectives

This protocol was designed as a cross-sectional population study of individuals reporting history of signature signs of loiasis, aiming to evaluate the prevalence of focal spleen lesions in individuals with *L*. *loa* infection living in highly endemic areas of Gabon. The cross-sectional study was followed by an observational longitudinal cohort study of the sub-group of subjects with spleen nodules on ultrasound, to observe the evolution of such focal lesions over time after routine treatment for loiasis (albendazole 400 mg twice a day for 21 days), as a secondary objective. Further secondary objectives were to evaluate the association of focal spleen lesions with circulating *L*. *loa* microfilariaemia; and to describe the presence of red blood cells (RBC) with Howell-Jolly bodies, as an indirect marker of hyposplenism, in infected participants.

### Study area

The study was performed between March-May 2023 (baseline evaluation) and December 2023-February 2024 (follow-up) in Ngounié, and Moyen-Ogooué provinces of Gabon, consisting of 80% rain forest, and known to be highly endemic for *L*. *loa* [5,18,19].

### Participant inclusion criteria and recruitment

Individuals of both sexes, living in the study area, aged 12 years and above and willing to provide written informed consent were invited to participate in the study.

Inclusion criteria encompassed reporting episodes of “eyeworm” migration in the past 12 months [20], and/or Calabar swelling, as assessed by interview. Specifically, the field team inquired, with the aid of a printed image of an eyeworm case, whether the potential participant noticed a worm moving on the white part of his/her eye and how long did the worm remained in the eye. For Calabar swelling, the interviewer asked whether the potential participant had experienced swelling in some parts of his/her body that appeared suddenly and disappeared a few hours or days later. Although these criteria excluded *L*. *loa*-infected individuals with no history of such clinical manifestations, and Calabar swelling is not as specific for loiasis since it has been described also in other infections such as in mansonellosis, these criteria were applied to maximize sensitivity for loiasis [21]. Exclusion criteria encompassed splenectomy, known pathologies which may confound the results of the study (including sickle cell disease, lymphoma, cancer, tuberculosis), history of noticeable weight loss in the past 12 months (which could reflect pathologies that may confound the results of the study), and treatment with IVM in the past 12 months. Conditional exclusion criteria were pregnancy (evaluated by last menstruation reported > 4 weeks before) and breastfeeding, due to the limitation of albendazole treatment in these conditions. Pregnant and breastfeeding women were not excluded a-priori from enrolment for the cross-sectional study but, in case of *L*. *loa* positivity, a pregnancy urine test was envisaged to be performed and treatment postponed after delivery and breastfeeding.

At the beginning of the study, the communities residing in the study regions were informed by the study staff in detail, in French, verbally and with the aid of written material, about the aims and procedures of this study as well as the benefits and risks, clinical and financial aspects, management of incidental findings, voluntary nature of participation and possibility for withdrawal at any time during the study. Field workers evaluated the eligibility of the potential participants and interested subjects/legal representatives of minors were requested to sign/fingerprint the ICF before taking part in the study.

### Study procedures

#### Blood sample analysis and categorization of participants

All enrolled individuals underwent withdrawal of 5 ml venous blood in potassium-EDTA during daytime (10 am– 3 pm) for the detection, identification, and quantification of circulating mf. Blood was transported within two hours from collection to the CERMEL laboratory and processed and examined by technicians trained and experienced in the assessment of blood slides, including mf morphology defining filarial species.

For mf detection, identification, and counting, two Giemsa-stained thick blood smears of 50 μL each were examined under a microscope and, if no mf were found, additional examination of 1 mL whole blood was performed after RBC lysis and centrifugation as a concentration technique. *L*. *loa* and *M*. *perstans* mf were identified based on morphological features including size, head, and tail characteristics, as per laboratory Standard Operating Procedures of CERMEL [5] and microfilaraemia (mf/ml blood) was computed. From the same blood sample, one thin blood smear was prepared, stained by Giemsa, and evaluated by microscopy for the presence of Howell-Jolly bodies as indirect marker of functional impairment of the spleen. All slides were screened for the presence of evident pathological levels of Howell-Jolly bodies [22]; a subset of slides from 112 participants (51%) were evaluated for quality purposes and quantification of Howell-Jolly bodies by two independent readers trained in the specific identification of these features. For quantification, 20 fields at 100X magnification were examined by each operator, the total number of RBC with Howell-Jolly bodies reported, and the mean between operators calculated. Percentage RBC with Howell-Jolly bodies was then calculated as the mean affected RBC per field divided by the mean number of RBC per field (standardized as 300).

Participants were categorized as follows: i) infected microfilaraemic with low microfilaraemia (<8000 mf/ml); ii) infected microfilaraemic with high microfilaraemia (≥8000 mf/ml); and iii) symptomatic amicrofilaraemic (eyeworm and/or Calabar swelling history in the past 12 months with no microfiaraemia). The definition of the latter group as “symptomatic amicrofilaraemic” rather than “occult loiasis” was chosen since the real *L*. *loa* infection status of the latter group could not be eventually ascertained clinically by direct observation of signs or through laboratory assays such as PCR. The threshold of 8000 mf/ml was chosen as clinically relevant since individuals with > 8000 mf/ml have an elevated risk of experiencing marked or severe adverse reactions upon treatment with IVM [4].

#### Ultrasonography and follow-up

All enrolled participants underwent abdominal ultrasound examination at CERMEL; transportation to and from CERMEL was free of charge for the participants. Ultrasound examination was performed using portable ultrasound machines (Ultrasound M-Turbo, Fujifilm Sonosite, Bothell, USA, or Mindray DP-30 Shenzhen, China), equipped with convex and liner probes. The ultrasonologist was blind to the infection status of the participant. Ultrasound exams were performed by a physician experienced in abdominal ultrasound (FT) or by physicians (BRA and FBM) trained in the evaluation of the spleen and standard views of the abdomen (transverse subcostal and subcostal views of left lobe, longitudinal section of the portal vein, oblique intercostal views of right liver lobe, and transverse and longitudinal views of the aorta). Images and video files of the spleen and abdominal scans were recorded for all participants, and reviewed for quality control by the experienced sonographer (FT), regardless of the live exam having been supervised or not.

The spleen was examined using convex and linear probes. Participants were examined in supine position, with the probe placed on the mid-axillary line, in the intercostal space. Size, margins, echotexture, as well as characteristics of any lesion, were recorded. Focal hypoechoic lesions were defined as “macronodules” if clearly detectable using a low-frequency (convex) probe (i.e., generally centimetric size or above), and as “micronodules” if clearly visible only with a high-frequency (linear) probe. Spleen size measurements were obtained on a longitudinal view passing through the spleen hilum. Splenomegaly was classified as mild (section area 45–60 cm^2^), moderate (>60–90 cm^2^), and severe (>90 cm^2^) [23]. In case of spleen abnormalities, the full abdomen was investigated with standard subcostal and longitudinal views for other pathological correlates, such as enlarged lymph nodes, or signs of portal hypertension in case of splenomegaly, or presence of focal liver lesions. After examination, participants were provided with a written report and addressed to the referral hospital for further medical consultations in case of findings potentially requiring medical attention but unrelated to loiasis.

Participants were treated free of charge for parasitic infections diagnosed during blood examination, following Gabonese national recommendations.

All enrolled participants with spleen nodules were re-assessed by ultrasound 6 months after treatment, if any was required.

### Sample size and data analysis

Based on an estimated prevalence of focal spleen lesions in individuals with *L*. *loa* infection of 5% in migrants from endemic areas [11], a samples size of 200 individuals with loiasis (microfilaraemic or amicrofilaraemic–as assessed by reporting specific clinical manifestations) was estimated to obtain a 95% confidence interval width of 6% for the point estimate.

Continuous variables like age and spleen size on ultrasound were summarized using median and interquartile range (IQR) while microfilaremia levels were summarized using geometric means (Gmean) and standard deviations (GSD) as well as median and IQR. All categorical variables (sex, splenomegaly, and presence of spleen nodules) were summarized with absolute and percentage frequencies.

Groups were compared using Chi-square or Fishers Exact test and Mann-Whitney U test as per variable scale. Analyses were carried out using R software version 4.2.3. A p-value <0.05 was considered statistically significant.

## Results

Between March and May 2023, 279 eligible people from the study region who reported eyeworm and/or Calabar swellings in the past 12 months were invited to participate in the study. Of these, 63 refused to undergo blood sampling and/or abdominal ultrasound, which led to 216 eligible participants with complete blood microscopy and abdominal ultrasonography for data analysis.

**Fig 1:**
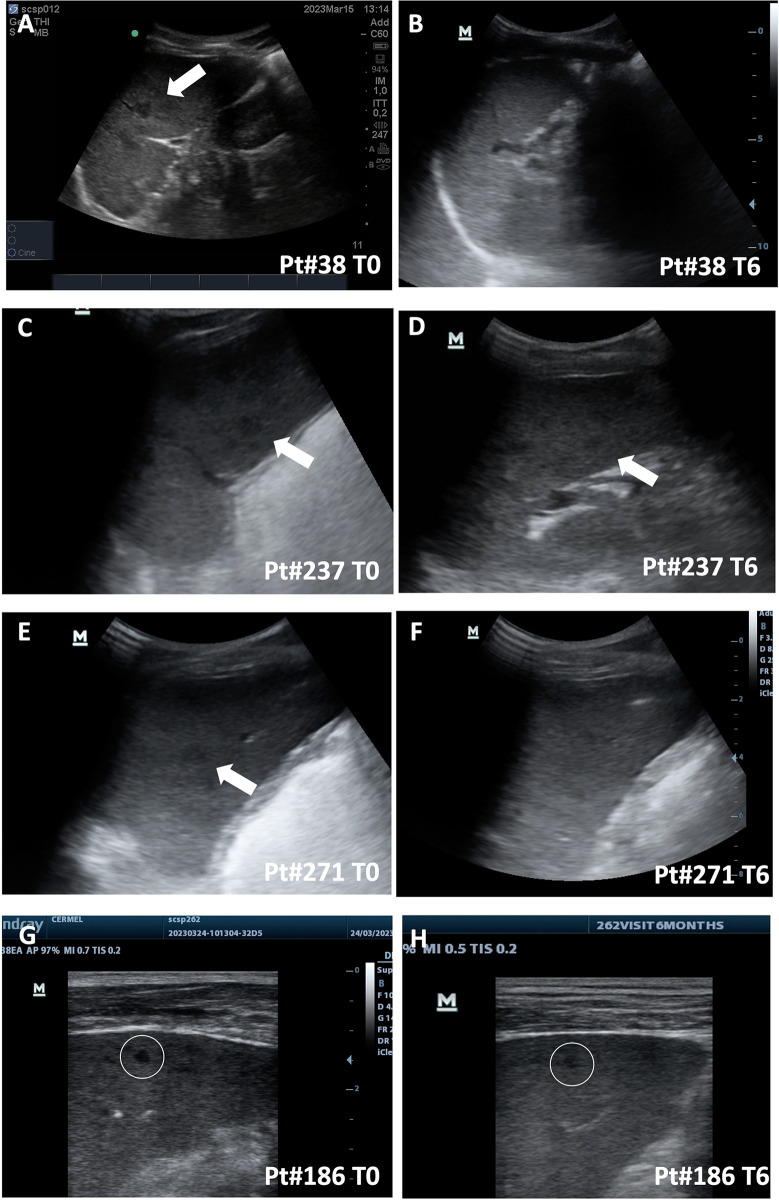
Representative static ultrasound views of focal spleen lesions at diagnosis (T0) and 6 months follow-up (T6). Videos of the ultrasound exams are available as S1–S14 Videos, to allow a better and comprehensive visualization of the lesions in the whole organ. Participant (Pt) identification code (Pt#n) corresponds to the code available at https://zenodo.org/records/13169677. Macro nodules shown in the static section are indicated by a white arrow; micronodules by a white circle **A, C, E]** participants with macronodules at T0, convex probe. **B, D, F]** 6 months follow-up ultrasound of participants with macronodules at T0, convex probe; note that modules are virtually disappeared in Pt#38 and Pt#271, while they are still visible in Pt#237. **G, H]** Representative T0 (G) and T6 (H) ultrasound of participant with micronodules, only visible with linear probe.

Demographic and clinical data of participants are summarized in Table 1; the complete data file is available at https://zenodo.org/records/13169677. Eighty-four participants were positive for *L*. *loa* mf (Gmean 115 mf/ml, GSD 22), and 132 were classified as infected amicrofilaraemic (108 reporting a history of eyeworm migration alone or in combination with Calabar swelling, and 24 only reporting episodes of Calabar swelling). Of these two groups, 12 (14.3%) and 11 (8.3%) had *M*. *perstans* infection as assessed by the presence of circulating *M*. *perstans* mf, respectively. On ultrasound examination, median spleen size was 42 cm^2^ (IQR 31–54), without statistically significant differences between groups for spleen size and prevalence of splenomegaly. No evident ultrasound signs of spleen fibrosis or evident hypotrophy were observed. Focal spleen lesions were observed in nine participants, of which, one was a small calcification, one abscess, one simple cyst, and in six cases were multiple macro- or micro-nodules. Multiple spleen macronodules, of approximately 1 cm size (Fig 1A and 1C and 1E, and S1–S3 Videos), were observed in three participants (1.4% of the entire cohort). All individuals with macronodules had microfilaraemic *L*. *loa* infection (2000, 2300, and 9958 mf/ml, the latter with *M*. *perstans* co-infection—50 mf/ml), equaling to a prevalence of 3.4% of participants with macronodules in the *L*. *loa* microfilaraemic group. Macronodules were well defined and hypoechoic on ultrasound.

Spleen hypoechoic micronodules (Fig 1G and S4–S6 Videos), observed only with the use of a liner probe, were detected in three participants (1.4% of the entire cohort), all of whom amicrofilaraemic for both *L*. *loa* and *M*. *perstans*, equaling to a prevalence of 2.3% micronodules in the amicrofilaraemic group. There was no co-existence of macro- and micro-nodules.

Participants with spleen macro- or micro-nodules had otherwise unremarkable abdominal ultrasound findings (normal spleen size or mild splenomegaly, no enlarged abdominal lymph nodes, normal liver ultrasonographical appearance) and did not report any symptom compatible with other infectious, inflammatory or neoplastic pathology which could have caused the observed focal spleen lesions [8].

At the 6 months US follow-up examination, the macronodules of two participants were no more visible (Fig 1B and 1F, and S7 and S9 Videos), while in the other participant the macronodules were unchanged (Fig 1D and S8 Video). Spleen micronodules were unchanged in all three participants at the 6 months follow-up (Fig 1H and S10–S12 Videos).

S13 and S14 Videos show representative spleen ultrasound exams of *L*. *loa* microfilaraemic individuals with low and high microfilaraemia, without spleen nodules.

No participant had malaria upon examination of blood smears. No evidently pathological levels of Howell-Jolly bodies were observed upon screening. This was confirmed by the results of quality check and Howell-Jolly bodies quantification carried out on the subset of slides from 112 participants. We found that at least one RBC with one Howell-Jolly body could be identified in 47 (42%) of these samples, but when considering even only these 47 samples with at least one RBC with one Howell-Jolly body, median affected RBC were 0.04% (range 0.01%-0.29%). These levels could be considered below clinical significance; indeed, a recent study carried out in Nigeria [22] found that the proportion of RBC containing Howell-Jolly bodies in healthy controls was 0.3% (IQR 0.1%-0.5%).

**Table 1:** Demographic and clinical characteristics of the enrolled participants. Only statistically significant p-values of differences between groups are reported in the table. GMean = geometric mean. GSD = Geometric standard deviation. IQR = interquartile range. mf = microfilariae. US = ultrasound. A^2^ = cross-section area. * borderline significant

	Total (N = 216)	Mf+ loiasis (N = 84)	High *L*. *loa* mf+ (N = 10)	Low *L*. *loa* mf+ (N = 74)	Symptomatic mf- (N = 132)	Significant p-values
**Age years—Median (IQR)** **[N missing data]**	48 (33, 59) 19	54 (40, 64) 18	51 (20, 63) 1	54 (40, 64) 17	44 (29, 56) 1	0.009 (Mf+ vs mf-) 0.007 (Low mf+ vs mf-)
**Sex–F (%): M (%)** **[N missing data]**	126 (64%): 72 (36%) 18	36 (54%): 31 (46%) 17	4 (44%): 5 (56%) 1	32 (55%): 26 (45%) 16	90 (69%): 41 (31%) 1	0.038 (Mf+ vs mf-)
***L*. *loa* microfilaraemia** **- GMean mf/ml (GSD)** **- Median mf/ml (IQR)**	-	115 (22) 120 (10–1955)	10,184 (2) 8,844 (8,844–9,694)	63 (16) 80 (8–742)	N/A	N/A
***M perstans* microfilaraemia** **- GMean mf/ml (GSD)** **- Median mf/ml (IQR)**	-	19 (4) 20 (16–52)	43 (2) 50 (35–65)	15 (5) 20 (4–40)	35 (4) 40 (20–55)	-
**US spleen size** **- Median A**^**2**^ **(IQR)**	42 (31–54)	39 (31–51)	45 (35–60)	37 (30–50)	43 (32–56)	-
**US splenomegaly** **- mild–N (%)** **- moderate–N (%)** **- severe–N (%)**	37 (17%) 32 (15%) 4 (1.9%)	15 (18%) 10 (12%) 1 (1.2%)	1 (10%) 2 (20%) 1 (10%)	14 (19%) 8 (11%) 0 (0%)	22 (17%) 22 (17%) 3 (2.3%)	-
**Presence of nodules** **- Macronodules–N (%)** **- Micronodules—N (%)**						
	3 (1.4%)	3 (3.6%)	1 (10%)	2 (2.7%)	0 (0%)	0.058 (Mf+ vs mf-)*
	3 (1.4%)	0 (0%)	0 (0%)	0 (0%)	3 (2.3%)	-

## Discussion

We evaluated spleen morphology, focal lesions, and spleen function, as assessed by the presence of Howell-Jolly bodies in peripheral erythrocytes as an indirect marker for functional impairment of splenic function, in participants with loiasis living in a highly endemic area in Gabon. We found that centimetric focal hypoechoic spleen lesions (macronodules) were present in about 3% of the microfilaraemic population with *L*. *loa* infection, confirming what observed previously in migrants with loiasis [8]. While we could not perform spleen biopsies to confirm the causal link between *L*. *loa* mf and spleen nodules, their morphology, prevalence and behavior, their presence in *L*. *loa*-mono infected individuals, together with the exclusion of people with confounding pathological conditions from our study, make these spleen lesions most likely caused by *L*. *loa*. Micronodules were not likely attributable to M. perstans infection either, since 2/3 were detected in M. perstans mf negative individuals, and no macronodules were observed in M. perstans only infected participants. The low prevalence of macronodules in a population highly endemic for loiasis is also compatible with their transitory nature. The precise incidence of spleen macronodules in individuals with loiasis of varying microfilaremia remains to be ascertained in future larger studies, as it remains unknown whether this phenomenon is physiological or occurring only in a subset of selected individuals/occasions. Also, it remains to be elucidated if treatment has any impact on the resolution of macronodules compared to their natural evolution, since for ethical reasons all parasitemic individuals were treated in our study. However, it is reasonable to assume that the evolution of macronodules would not be influenced by treatment, since they appear to derive from the granulomatous reaction around already dead/dying microfilariae [10,12,13,24–26]. Furthermore, in one out of three participants with spleen macronodules, these did not change after treatment for loiasis, supporting the hypothesis that treatment had no influence on the evolution of spleen granulomas. Based on the observation that macronodules may disappear over time without morphological alteration of the spleen, the lack of evident markers of functional impairment in patients with splenic lesions and the lack of other observed pathologies associated to this morphological finding, all seem to point towards the interpretation of transient formation of macronodules as being a rather benign phenomenon in terms of impact on spleen morphology and function. Although spleen size was measured in terms of section area rather than volume, this is a widely used parameter [23], which, together with qualitative assessment of morphology (margins, echotexture) could confidently be used to evaluate spleen structure.

The correlation between levels of *L*. *loa* microfilaraemia and occurrence of macronodules is difficult to ascertain from the results of this study; while the point estimate of 10% prevalence in high microfilaremic loiasis as opposed to 2.7% in low microfilaremic individuals might suggest a causal relationship of high levels of circulating mf with the presence of spleen nodules, the sample size was small and the difference was not statistically significant. The absence of macronodules in amicrofilaraemic individuals reporting a history of eyeworm and/or Calabar swelling could be interpreted in different ways. Indeed, it must be taken into consideration that the real infection status of this group remains unknown and their supposed infection with *L*. *loa* relied only on what reported by the participants themselves. Therefore, it is possible that either macronodules indeed form only in individuals with detectable *L*. *loa* microfilaraemia, or that they also form in individuals with occult loiasis (i.e. infected with *L*. *loa* but without circulating mf at a microscopic level), but the latters were only a fraction of those included in our “symptomatic amicrofilaraemic” group (the other fraction being formed by people with symptoms due to other conditions, e.g., *M*. *perstans* infection) and the size of this subgroup was too small to detect any spleen nodules. However, if we consider that over 80% of the “symptomatic amicrofilaraemic” group in this study reported a history of eyeworm, a clinical manifestation much more specific for loaisis than Calabar swelling, we can reasonably assume that the majority of this group was actually composed of participants with loiasis and therefore that indeed macronodules occur in the presence of *L*. *loa* microfilaraemia, and not, or more rarely, in occult loiasis. The further hypothesis that occult infection (i.e., absence of microfilaraemia) could be actually caused by a particularly active removal of mf from blood by the spleen seems also unlikely since it could be assumed that this active removal of mf would be associated with higher incidence (and therefore point prevalence) of macronodules in this group, which was not the case in our study.

In addition to macronodules, we could observe spleen micronodules in about 1.4% of the whole examined cohort, all in participants with no circulating mf of either *L*. *loa* or *M*. *perstans*. These micronodules seemed to have a different behavior over time compared to macronodules, since all were still present, unchanged, after 6 months. If we assume that in some *L*. *loa* microfilaraemic individuals dead/dying mf induce spleen granulomas objected by the presence of macronodules but not micronodules on ultrasound, and that micronoduels were not likely attributable to M. perstans infection either, the origin of micronodules in our cohort remains unclear. Spleen micronodules, visible only with the use of a high-frequency liner ultrasound probe, have been described in pathological conditions such as lymphoma, sarcoidosis, extrapulmonary tuberculosis, and low-CD4+ HIV [27,28], but also in healthy children and adolescents [29]. Although we could not carry out an exhaustive clinical and laboratory evaluation of our participants with spleen micronodules, clinical history (all participants with the possible conditions listed above) and otherwise unremarkable abdominal ultrasound findings seemed excluding the presence of major evident pathologies in this group. The absence of observation of spleen micronodules in participants with *L*. *loa* microfilaraemia is intriguing, but the spleen is an organ with a complex architecture [28], and the prevalence of a micronodular spleen pattern in the general population is unknown, therefore it is difficult to speculate on a possible link (or lack thereof) with the occult loiasis status.

Our study had several limitations. We included only participants who reported signs and symptoms of loiasis (history of eyeworm migration and/or Calabar swelling) in the previous 12 months and we did not carry out PCR on blood for *L*. *loa* infection. This selection was required due to financial and logistic constraints, that did not allow us to perform the study procedures on a larger sample of individuals. Therefore, it is possible that a proportion of participants were potentially misclassified and we could not formally evaluate the prevalence of spleen lesions in asymptomatic *L*. *loa*-infected subjects, and in the general population. However, we could confirm a similar prevalence and behavior of macronodules in individuals residing in endemic regions with symptomatic, microfilaraemic loiasis as observed in *L*. *loa* infected migrants in a non-endemic setting [8]. This, together with the observation that the presence of spleen macronodules does not appear to be correlated with level of microfilaraemia, suggests that the macronodules prevalence found in this study could reflect that of the general *L*. *loa*-infected population. Furthermore, our results seem pointing towards an overall normal spleen function in individuals with loiasis, although we did not carry out a more advanced evaluation using different immunological, haematological and scintigraphic parameters [30]. A further limitation was the impossibility to evaluate the natural history of the spleen macronodules in the absence of treatment. However, as detailed above, since albendazole is not microfilaricidal and spleen lesions appear to form around already dead microfilariae, any influence of treatment on nodules evolution seems unlikely. Finally, we excluded from our study participants with signs (e.g., fever) or reporting symptoms compatible with pathologies (e.g., TB) that could have biased our results as known to cause spleen lesions. Therefore, we could have excluded a fraction of the population where *L*. *loa* infection was associated with higher risk of infectious diseases, and therefore we could have missed a higher frequency of spleen alterations due to loiasis in this group. The assessment of such population would have required both a different recruitment (e.g, in hospitals) and study design (e.g., longitudinal study with in-depth differential diagnostic and treatment capacity), which was not possible here. However, we could assume that a fraction of this *L*. *loa*-infected population, supposing more prone to acquiring infectious diseases, would be included in our cohort, because in an asymptomatic period, and that this would have shown features of spleen damage, if the spleen was actually the reason of their increased incidence of infections. Therefore, our findings suggest that the transient macronodules forming in *L*. *loa* infection are most likely not causing overt spleen hypo- or dysfunction.

To conclude, spleen centimetric hypoechoic macronodules are present in a small but consistent proportion of individuals with microfilaraemic loiasis, without obvious impact on spleen function. The occurrence of such focal spleen lesions should be taken into consideration by physicians (especially radiologists and infectious disease specialists) when investigating differential diagnoses of focal spleen lesions to avoid misdiagnosis and mistreatment. Further studies should expand the examination of spleen morphology and function to other groups excluded in this study, ideally using a longitudinal study design and postponing treatment for loiasis, to confirm the generalizability of our results. In the same view, it would be also be worth evaluating the prevalence and behavior over time of spleen micronodular ultrasound patterns in the general population.

## Supporting information

S1 Video Spleen scan with convex probe of participant #38 at first examination, showing hypoechoic macronodules. (MP4)

S2 Video Spleen scan with convex probe of participant #237 at first examination, showing hypoechoic macronodules. (AVI)

S3 Video Spleen scan with convex probe of participant #271 at first examination, showing hypoechoic macronodules. (AVI)

S4 Video Spleen scan with linear probe of participant #151 at first examination, showing hypoechoic micronodules. (AVI)

S5 Video Spleen scan with linear probe of participant #186 at first examination, showing hypoechoic micronodules. (AVI)

S6 Video Spleen scan with linear probe of participant #234 at first examination, showing hypoechoic micronodules. (AVI)

S7 Video Spleen scan with convex probe of participant #38 at 6 months follow-up, showing virtual disapperarance of the hypoechoic macronodules. (AVI)

S8 Video Spleen scan with convex probe of participant #237 at 6 months follow-up, showing persistence of the hypoechoic macronodules. (AVI)

S9 Video Spleen scan with convex probe of participant #271 at 6 months follow-up, showing disapperarance of the hypoechoic macronodules. (AVI)

S10 Video Spleen scan with linear probe of participant #151 at 6 months follow-up, showing persistence of the hypoechoic micronodules. (AVI)

S11 Video Spleen scan with linear probe of participant #186 at 6 months follow-up, showing persistence of the hypoechoic micronodules. (AVI)

S12 Video Spleen scan with linear probe of participant #234 at 6 months follow-up, showing persistence of the hypoechoic micronodules. (AVI)

S13 Video Spleen scans with convex probe of microfilaraemic participant #32, having low *L*. *loa* microfilaraemia, showing normal morphology. (MP4)

S14 Video Spleen scans with convex probe of microfilaraemic participant #164, having high *L*. *loa* microfilaraemia, showing normal morphology. (MP4)
